# Utilization of colorectal cancer screening tests across European countries: a cross-sectional analysis of the European health interview survey 2018–2020

**DOI:** 10.1016/j.lanepe.2024.100920

**Published:** 2024-04-29

**Authors:** Idris Ola, Rafael Cardoso, Michael Hoffmeister, Hermann Brenner

**Affiliations:** a Division of Clinical Epidemiology and Aging Research, German Cancer Research Center (DKFZ), Heidelberg 69120, Germany; b Medical Faculty Heidelberg, University of Heidelberg, Heidelberg 69120, Germany; c Division of Preventive Oncology, German Cancer Research Center (DKFZ) and National Center for Tumor Diseases (NCT), Heidelberg 69120, Germany; d German Cancer Consortium (DKTK), German Cancer Research Center (DKFZ), Heidelberg 69120, Germany

**Keywords:** Colorectal cancer, Cancer screening, Colonoscopy, Fecal test, Cancer epidemiology, Cancer prevention and control

## Abstract

**Background:**

Colorectal cancer (CRC) screening has been shown to reduce CRC incidence and mortality, and most European countries have started to implement CRC screening programs in the past 20 years. Consequently, this study aimed to estimate the utilization of fecal tests and colonoscopy, as well as investigate factors associated with their utilization based on specific screening program characteristics in European countries.

**Methods:**

We analyzed data from the European Health Interview Survey 2018–2020 to determine the utilization of fecal tests [guaiac-based fecal occult blood test (gFOBT) or fecal immunochemical test (FIT)] within the preceding 2 years or colonoscopy within the preceding 10 years among people aged 50–74 years, based on the type of screening offered in each country. Using multivariable logistic regression and sub-group meta-analysis, factors associated with screening use were determined.

**Findings:**

The analyses included data from 129,750 respondents across 29 European countries, with participant counts ranging from 1511 individuals in Iceland to 11,755 individuals in Germany. Unit response rates ranged from 22% to 88%. The use of either test was highest among countries with fully rolled-out programs with fecal tests [from 37.7% (867/2379) in Croatia to 74.9% (2321/3085) in Denmark] and in countries offering colonoscopy as an alternative screening method [from 26.2% (854/3329) in Greece to 75.4% (1192/1760) in Luxembourg]. We observed the lowest utilization of either test in countries with no program or small-scale programs [6.3% (195/3179) in Bulgaria to 34.2% (722/2144) in Latvia]. Across all types of screening offers, younger age, being without a partner, low education, rural residence, and living in large households were associated with lower utilization, as were poor lifestyle scores and prolonged periods without physician consultation.

**Interpretation:**

Our findings point to large disparities and much room for improvement in CRC screening offers and utilization across Europe.

**Funding:**

There was no funding source for this study.

**Research in context**

**Evidence before this study**

Over the past two decades, many European countries, particularly those with long-running colorectal cancer (CRC) screening programs, have witnessed a notable decline in both CRC incidence and mortality rates. Despite these positive trends, the utilization of screening tests remains suboptimal across many European countries.

An earlier investigation revealed significant variation in the adherence of the general eligible population to colonoscopy or fecal tests across European countries, ranging from 6% to 71%. Our literature search, conducted through PubMed from 2003 until May 30, 2023, utilized search terms such as ‘colorectal cancer’ combined with ‘fecal occult blood test,’ ‘faecal immunochemical test,’ ‘colonoscopy,’ AND ‘population-based screening’ or ‘mass screening.’ Additionally, manual searches were performed on the websites of cancer registries and health ministries. Our investigation revealed a significant expansion of population-based screening programs across many European countries. This prompted a critical analysis of screening utilization, with the aim of evaluating the progress made towards meeting the screening offering and utilization targets set by the EU Commission for 2025.

Here, we evaluated fecal tests and colonoscopy use, as well as factors associated with usage in Europe, utilizing data from the latest (third) wave of the European Health Interview Survey (2018–2020), a large population-based survey from 31 countries in Europe.

**Added value of this study**

Our study reveals progress in CRC screening in Europe, although only a few countries still have the majority of the eligible population up-to-date with screening. Overall, organized, fully rolled-out screening programs with fecal tests achieved the highest utilization of the screening tests, as did those offering colonoscopy as an alternative screening modality. Across all types of screening offers, people with various indicators of low socioeconomic status and those with unhealthy lifestyles and the highest CRC risk demonstrated the strongest underuse of CRC screening tests.

**Implications of all the available evidence**

Our study provides policy-relevant insights into the country-level performance of CRC screening across Europe, particularly in relation to the type of screening programs offered. The results may inform efforts towards attaining the EU's CRC screening targets, and to avert the projected rise in CRC incidence and mortality attributed to demographic shifts anticipated in the decades ahead.

## Introduction

Colorectal cancer (CRC) is the third most common cancer worldwide and accounts for about 10% of the nearly 19.3 million new cancer cases diagnosed annually.^1^ An estimated 520,000 new CRC cases occurred in Europe in 2020, about 95% of which occurred among people 50 years of age or older.^2^ In the European Union (EU), an estimated 12.4% of all cancer mortality is due to CRC.^3^

In 2003, the EU created an ambitious cancer screening plan with the objective of offering screening to 90% of the eligible European population for specific cancers, including CRC, by 2025 through improvements in accessibility and quality of screening as well as diagnosis.^4^ Over the past 20 years, incidence and mortality rates for CRC have decreased in several European countries with long-running screening programs.^5^ Several of these programs incorporate fecal testing, including guaiac-based fecal occult blood tests (gFOBT) or fecal immunochemical tests (FIT), as well as colonoscopy. The delivery strategy for these screening methods varies, encompassing organized programs and opportunistic approaches.^6^, ^7^, ^8^

In a previous analysis of data from the second wave (2013–2015) of the European Health Interview Survey (EHIS)^6^ we demonstrated that the proportion of the general eligible population that was up-to-date with either of these screening methods varied considerably across European countries (from 6% to 71%). We also noted that younger eligible individuals, those who had not been to the doctor recently, and those at increased risk for CRC based on their lifestyle were less likely to report having undergone either of these tests.^6^

In the last decade, population-based CRC screening programs have been further rolled out in several European countries, and analyses of screening use for more recent years would be very informative and relevant to assess achieved levels of screening utilization. Using data from the third wave of EHIS (2018–2020), this study aimed to estimate fecal test and colonoscopy use as reported in the EHIS 2018–2020 and examine factors associated with usage in European countries. In addition, the insights gleaned from this study, in conjunction with findings from EHIS-2 and other previous studies,^6^, ^7^, ^8^ can provide an opportunity for understanding the changes made in screening coverage and test utilization since previous analyses.

## Methods

### Study design and data source

A cross-sectional study was conducted using data from EHIS-3. EHIS is a nationally representative population-based statistical survey on health status, health determinants, and healthcare utilization aimed at supporting policies addressing health inequalities, social exclusion, and healthy aging in the European Union.^9^ The survey targets non-institutionalized individuals 15 years of age and older who are residents of each included country at the time of the survey. According to Commission Regulation (EU) No. 2018/255, EHIS-3 was carried out in 2018–2020 in 31 countries, including all EU member states as well as Iceland, Turkey, Norway, and Serbia.^9^ The participating countries built their sampling frames using a variety of data sources, including population censuses, population and housing registrations, and additional sources including the Register of Census Districts and Buildings in the Czech Republic and the Telephone Database in Germany.^9^ For this study, the anonymized microdata (individual-level data) provided by each participating country was used.

### Data collection

Survey instruments were mostly interviewer-administered and varied among countries, but were generally based on questionnaires and interviews administered face-to-face, via telephone, by mail, or by a varying combination of these modalities. Only Denmark, Finland, Luxembourg, and Sweden adopted questionnaires that were completely self-administered via online or paper-based forms. The unit response rate exceeded 60% in 15 countries, but ranged from 22% in Germany to as high as 88% in Romania.^9^ Data collection took 3–18 months in various countries, with an average interview duration of 20–67 min per respondent.^9^ Other details about the EHIS survey have been described in the Quality Report for EHIS 3.^9^

### Inclusion/exclusion criteria and sample size

Men and women aged 50–74 years are typically considered eligible for CRC screening in accordance with EU Council Recommendation^4^ and were included in our analysis. To limit potential reporting errors, data obtained by proxy interviews were excluded, and only data provided by actual respondents were used in the analysis.^6^

A total of 129,750 respondents were included in our analysis after excluding respondents from France (14,192), whose microdata were not released for our study, proxy interviews (14,544), and respondents outside of the recommended age range (167,091).

**Fig. 1:**
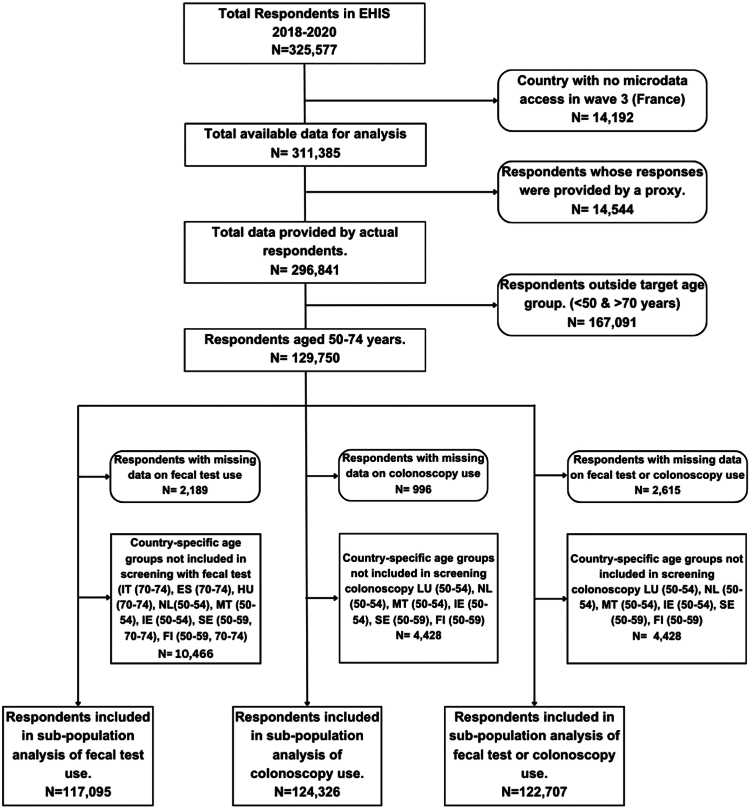
Study flowchart showing the respondents included in the final analyses. Abbreviations: IT, Italy; ES, Spain; HU, Hungary; NL, The Netherlands; MT, Malta; IE, Ireland; SE, Sweden; FI, Finland; LU, Luxembourg.

After excluding missing data and age groups not included in screening programs in some countries, a subtotal of 117,095, 124,326, and 122,707 respondents were included in the analysis of the utilization of fecal test, colonoscopy, and either test, respectively (Fig. 1).

### Measures

#### Individual-participant measures

Information on any colonoscopy use in the preceding 10 years, any fecal tests (gFOBT or FIT) use in the preceding two years, and use of any or both of the two tests in the specified time periods was obtained by asking the participants whether and when was the last time these tests were done. These time periods are the recommended and most used screening intervals in most European countries.

To assess fecal test utilization, the respondents were asked, “*When was the last time you had a faecal occult blood test?*” with possible responses as follows: *(1) Within the past 12 months (2) 1 to less than 2 years (3) 2 to less than 3 years (4) 3 years or more (5) Never*.^10^ Colonoscopy use was assessed with the question “*When was the last time you had a colonoscopy?*” with possible responses as follows: *(1) Within the past 12 months (2) 1 to less than 5 years (3) 5 to less than 10 years (4) 10 years or more (5) Never*.^10^ To ensure accuracy in the responses, interviewers were allowed to, when necessary, adapt the questions and add important clarifications, such as outlining the procedure and goals of each test for the respondent.^10^ Information about use of other screening tests such as sigmoidoscopy, CT colonography, or fecal DNA tests were not provided in the EHIS survey.

The Andersen behavioral model provided the theoretical framework for the analysis of the potential determinants of CRC screening test utilization.^11^ The model is based on three dimensions of predisposing characteristics: *demographic factors*, including sex and chronological age; *enabling factors*, such as household income; and *need variables*, including self-rated health status.^11^ Information fulfilling the model’s three dimensions was provided in the EHIS modules, namely socioeconomic and demographic variables (age [50–74 years], sex, education, employment status, location of residence, citizenship, income level, and household size), healthcare utilization variables (last medical or surgical consultation and hindrance to health care access), and health status variables (self-perceived health, health-related limitation, mental health status, and healthy lifestyle score).^5^^,^^9^

The derivation of the healthy lifestyle score (HLS), which was originally proposed by Carr and colleagues,^12^ has been described in detail in a previous study.^6^ Briefly, factors known to lower CRC risk were dichotomized, with each respondent assigned one point for each of the following low-risk behaviors: not smoking regularly; physical activity of at least 150 min of moderate-intensity or 75 min of high-intensity recreational or sporting activities per week; a body mass index within the normal range (18.5–25 kg/m^2^); and no or only moderate alcohol consumption of less than two alcoholic drinks daily.^6^^,^^12^ The dietary component of the HLS could not be derived from the EHIS data. Therefore, the HLS was calculated based on four lifestyle factors, with values ranging from 0 (least healthy) to 4 (most healthy) (Supplementary Table S1).

#### Country-level measures

The CRC screening programs in the 31 EHIS-participating countries were reviewed, and countries were classified by type of CRC screening offer following the method adopted in our previous analysis,^6^ as outlined in detail below. Websites of national and regional cancer registries, health ministries and agencies, and a literature search of recent articles in PubMed provided information that aided the classification (Supplementary Table S2).

Fecal test utilization was analyzed by stratifying the EHIS-participating countries into four major categories based on the types of programs and level of coverage at the time of data collection for EHIS-3 (2018–2020).^6^^,^^9^

A. Countries with organized programs that have been fully implemented nationwide using fecal tests.
B. Countries with organized programs that were implemented only partially or in some regions using fecal tests.
C. Countries offering fecal testing in opportunistic programs.
D. Countries with only a small-scale pilot program or no program using fecal tests.

Four different categories were adopted to guide the analysis of colonoscopy use, given the fact that few countries in Europe offer colonoscopy as a primary screening exam.

A. Countries with organized programs that have been fully implemented nationwide using fecal tests.
B. Countries with organized programs that were implemented only partially or in some regions using fecal tests.
C. Countries where colonoscopy is being offered as an alternative CRC screening modality.
D. Countries with no program at all, small-scale organized programs, or opportunistic programs with fecal tests.

### Statistical analyses

In all analyses, survey weights, derived by calibration methods and described in detail in the EHIS 3 methods,^9^ were applied to each respondent to reduce potential non-response bias and to ensure that each country is represented in proportion to its demographic distribution. The participants’ socioeconomic and demographic characteristics and the utilization rates of the different types of tests by groups of countries (as outlined above) and individual countries were descriptively analyzed.

Multivariable logistic regression models were used for inferential statistics on factors associated with screening use on the individual level. Odds ratios (OR) and their 95% confidence intervals (CIs) were derived as measures of the association between a number of factors, including sociodemographic variables, the HLS and indicators of healthcare use, and the usage of FOBT within the last two years and/or colonoscopy within the last 10 years. Weighted estimates for the ORs based on the complex survey design were computed using the Horvitz-Thompson estimator method. After obtaining the ORs and associated robust standard errors for each country, subgroup meta-analyses using the Mantel-Haenszel method were performed with the meta package in RStudio Version 4.3.2 (RStudio, Inc., Boston, MA, USA) to determine the ORs and CIs by type of screening offer. To assure the validity of our statistical findings, robust standard errors were used to account for both the potential heteroscedasticity in the residuals and the sample weights allocated to each observation.^13^

All analyses were performed using RStudio software, with figures (except Fig. 1) generated using the ggplot2 package within RStudio. All tests were 2-sided, with a p-value of <0.05 taken as statistically significant. Results are presented in text, tables, and figures, and all reported estimates are weighted.

### Legal and ethical authorization

The “Regulation (EC) No 1338/2008 of the European Parliament and of the Council of 16 December 2008 on Community Statistics on Public Health and Health and Safety at Work (Text with EEA Relevance)" provided the legal authorization for EHIS surveys.^14^ According to Commission Regulation (EU) No. 2018/255, each country’s data collection methods and data collected are outlined. The institutions in charge of conducting the survey in each participating country received ethical clearance at the national level. Access to EHIS wave 3 microdata was granted by Eurostat with proposal number RPP 294/2022-EHIS.

### Role of the funding source

There was no funding source for this study.

## Results

Table 1 provides an overview on main study population characteristics. A slight majority of participants were female, and the proportion of participants in five-year age groups slightly decreased from 23.8% in age group 50–54 to 15.2% in age group 70–74. Approximately two thirds of participants were married and lived in households with up to two persons, slightly less than half of participants were (still) employed.

**Table 1:** Sociodemographic characteristics of the study population.

Characteristics	N (Unweighted %) (Total = 129,750)	Weighted %
Sex		
Male	59,571 (45.9)	47.5
Female	70,179 (54.1)	52.5
Age group (years)		
50–54	25,905 (20.0)	23.8
55–59	26,704 (20.6)	21.6
60–64	27,402 (21.1)	21.1
65–69	26,838 (20.7)	18.3
70–74	22,901 (17.7)	15.2
Marital status		
Married/registered partners	86,026 (66.5)	65.8
Never married	14,136 (10.9)	11.7
Widowed/Divorced	29,295 (22.6)	22.5
Educational level^a^		
Tertiary education	32,995 (25.6)	22.3
Upper secondary level	56,200 (43.5)	47.4
No education or less than upper secondary level	39,956 (30.9)	30.3
Residence		
City	41,647 (33.6)	36.5
Town or suburbs	41,449 (33.5)	36.4
Rural	40,704 (32.9)	27.2
Employment status^b^		
Employed	53,574 (41.5)	45.8
Unemployed and others	21,741 (16.8)	17.4
Retired	53,815 (41.7)	36.8
Income level^c^		
Quintiles 4 and 5	52,650 (42.9)	44.3
Quintile 3	25,258 (20.6)	19.8
Quintiles 1 and 2	44,829 (36.5)	35.9
Citizenship^d^		
Native born	122,143 (96.5)	96.7
Non-natives	4435 (3.5)	3.3
Household size		
<2 people	89,695 (69.3)	65.8
≥3 people	39,798 (30.7)	34.2

a Educational level was based on the highest level of education completed based on ISCED classification. Tertiary level includes Short-cycle tertiary education, bachelor’s or equivalent level, Master’s or equivalent level, and Doctoral or equivalent level. Secondary level includes Upper secondary education and post-secondary non-tertiary education and No education or less than upper secondary level includes: No formal education or below ISCED 1, Primary education, and Lower secondary education.

b Unemployed and others category include those that are unemployed, unable to work due to longstanding health problems, student, pupil, fulfilling domestic tasks, on compulsory military or civilian service, and others not classified.

c Based on self-reported disposable household income.

d Non-native category include respondents born in another EU Member State and in a non-EU country.

### CRC screening offers in the EU countries, Iceland, Norway, and Serbia

Table 2 presents a summary of the relevant characteristics of CRC screening programs in the EHIS-participating countries. Most countries have adopted an organized structure for their screening programs, although considerable variations still exist in the target age group, population coverage, and type of screening tests. Among countries with organized screening programs with fecal tests, six had fully rolled out their programs nationwide (Denmark, the Netherlands, Slovenia, Belgium, Lithuania, and Croatia), whereas 10 countries had only covered a fraction of the eligible groups or had the screening programs running in a few regions (Czechia, Portugal, Malta, Italy, Ireland, Sweden, Spain, Hungary, Finland, and Serbia). Opportunistic screening, mostly with fecal tests, was also offered in five countries, including Austria, Germany, Slovakia, Latvia, and Greece. Only five countries (Luxembourg, Austria, Germany, Iceland, and Greece) offered colonoscopy as a primary screening modality. The remaining countries either did not have any screening programs or were running only a small-scale pilot program.

**Table 2:** Classification of EHIS-participating countries by type of CRC screening programs.

Country	Structure of screening program	Year of program initiation (& possibly, termination)	Age group	Type of screening test	Screening interval	Reference^h^	Category according to gFOBT/FIT offer
Belgium (Flemish region)	Organized	2013–2018	56–74	FIT	2 yrs	1,2	A
		2018–2020	53–74				
		2020	50–74				
Belgium (Wallonia/Brussels)		2009–2016	50–74	gFOBT			
		2016		FIT			
Croatia	Organized	2007	50–74	gFOBT	2 yrs	3–5	
Denmark	Organized	2014	50–74	FIT	2 yrs	6	
Lithuania (Vilnius and Kaunas)	Organized	2009–2014 (pilot)	50–74	FIT	2 yrs	7,8	
Lithuania (All regions)		2014					
The Netherlands	Organized	2014	55–75	FIT	2 yrs	9	
Slovenia	Organized (pilot)	2008–2009	50–69	FIT	2 yrs	10,11	
	Organized	2009–2015	50–74				
		2015					
Czechia	Organized (pilot)	1979–1998	45–60	gFOBT	2 yrs	12–15	B
	Opportunistic	2000–2009	50–54				
	Opportunistic	2009	50–54	FIT/Colo	1 yr		
	Opportunistic	2009	55+	FIT/Colo	2 yrs/10 yrs		
	Organized	2014	50+	FIT	2 yrs		
Finland^a^	Organized	2004–2016	60–69	gFOBT	2 yrs	16,17	
	Organized (pilot)	2019–2027	56–74	FIT			
Hungary (Csongrád county)	Organized	2013–2015	50–70	FIT	2 yrs	18,19	
Hungary^b^	Organized	2018					
Ireland	Organized	2012	55–74	FIT	2 yrs	3,20,21	
Italy	Organized	1982–1996	50–69	gFOBT	2 yrs	22–24	
	Organized	1996	50-70/74	FIT			
Italy (Piedmont and Veneto regions)	Organized	2003/2004	58–60	FS	Once only		
Malta	Organized	2012	55–66	FIT	2 yrs	5,25	
		NR	55–74		2 yrs		
Portugal	Opportunistic	NR	50–74	FIT/Colo	1 yr/10 yrs	12,26–28	
Portugal (Alentejo and Central regions)	Organized	2009–2018	50–70	gFOBT	2 yrs		
		2018		FIT			
Portugal (Northern region)	Organized (pilot)	2016–2018	50–74	FIT			
	Organized	2018					
Serbia (32 municipalities)	Organized	2013–2014	50–74	FIT		29,30	
Spain	Organized	2000–2010	50–69	gFOBT	2 yrs	3,12,31–33	
		2010		FIT			
Sweden (Regions Gotland and Stockholm)	Organized	2008–2015	60–69	gFOBT	2 yrs	5,24,34	
		2015		FIT			
Sweden (Region Skåne)		2021	60–74	FIT			
Austria (Burgenland)	Organized	2003	40–80	FIT	1 yr	24,35	C
Austria (Vorarlberg)^c^		2007	50+	Colo	10 yrs		
Austria (All regions)	Opportunistic	1980	40+	gFOBT,	1 yr		
		2005	50+	Colo	7–10 yrs		
Germany	Opportunistic	1977–2002	45+	gFOBT	1 yr	36–38	
	Opportunistic	2002–2017	50–54	gFOBT	1 yr		
		2017		FIT			
	Opportunistic	2002–2017	55+	gFOBT/Colo	2 yrs/10 yrs		
		2017		FIT/Colo			
	Organized	2019	50-54/55+	FIT	1 yr, then 2 yrly		
	Organized	2019	50+(men); 55+(women)	Colo (up to 2 screenings)	10 yrs		
Greece	Opportunistic	NR	50–74	gFOBT	NR	3,33	
				Colo	NR		
Latvia	Opportunistic	2005	50–74	gFOBT	1 yr	3,39	
Slovakia	Opportunistic	2002–2019	45–75	gFOBT/Colo	2 yrs	40	
	Organized (pilot)	2019–2021	50–75	FIT			
	Organized	2021		FIT			
Bulgaria^d^	Opportunistic	2009	NR	gFOBT	NR	41	D
	No program	2009		NA	NA		
Cyprus^e^	No program	NA	NA	NA	NA	42	
Estonia^f^	Organized (Pilot)	2016	60–69	FIT	2 yrs	43,44	
	Organized	2022					
Iceland	Opportunistic	NR	50+	Colo	NR	45	
Luxembourg	Opportunistic	2005-	50+	gFOBT/Colo	NA	12,46	
	Organized (Pilot)	2016–2019	55–74	FIT/Colo	2 yrs/10 yrs		
	Organized	2021		FIT	2 yrs		
Norway (Østfold, Akershus & Buskerud Counties)	Organized (pilot)	2012–2018	50–74	FS/FIT	2 yrs (FIT), Once (FS)	47–50	
Norway (All regions)^g^	Organized	2022	55+	FIT	2 yrs		
Poland	Opportunistic	2000–2011	50–66	Colo	10 yrs	51–53	
Poland (25 of 380 counties)	Organized (pilot)	2012	55–64	Colo	Once only		
Romania	No program	NA	NA	NA	NA	54	

The countries are arranged in alphabetical order within each category based on the type of CRC screening programs offered with regards to fecal tests. Category A, countries with nationwide organized screening fully implemented using fecal tests; Category B, countries with organized programs with fecal tests partially rolled out or with regional coverage only; Category C, countries with opportunistic programs with fecal test; category D, countries with no program with fecal tests or a small-scale pilot program only.

gFOBT, guaiac-based fecal occult blood test; FIT, fecal immunochemical test; FS, flexible sigmoidoscopy; Colo, colonoscopy; NA, not applicable; NR, not reported; yr(s), year(s).

a In Finland, following the pilot of FIT in 2019–2021 in volunteer municipalities, the Government Decree on Screenings was amended to make screening available nationwide from 2022 onwards. Screening every 2 years was initially introduced among 60–68-year-olds and will be expanded to all target age groups (56–74) by 2031 (https://cancerregistry.fi/screening/colorectal-cancer-screening/).

b In Hungary, eligible persons are invited by the National Public Health Institute based on their association with GPs who are participating in the screening program. The GPs are reimbursed per screened individual (Supplementary Item S6, ref. 19).

c In Vorarlberg, screening is only available for insured individuals according to the protocol (Supplementary Item S6, ref. 24).

d In Bulgaria, general practitioners (GPs) were responsible for the referral of patients for gFOBT until 2009, when the opportunistic program was discontinued due to poor compliance by both the practitioners and the eligible persons (Supplementary Item S6, ref. 41).

e In Cyprus, a small-scale pilot program was said to have been conducted in 2013; however, no further information has been provided about the continuation of any screening program since then.

f In Estonia, an organized screening program was piloted among insured persons from 2016 until 2022, when it was expanded to all eligible individuals.

g In Norway, organized screening with FIT was piloted between 2012 and 2018 in Østfold, Akershus, and Buskerud Counties. Since May 2022, the Norwegian Cancer Registry has administered a nationwide CRC screening with FIT every 2 years from age 55. The program is expected to be replaced with a once-only screening colonoscopy after five FIT screening rounds, when colonoscopy capacity would have improved to cover the entire target population (Supplementary Item S6, ref. 47).

h Reference list for Table 2. See Supplementary Item S6 in the Supplementary document.

### CRC screening utilization rates by type of CRC screening offer

**Fig. 2:**
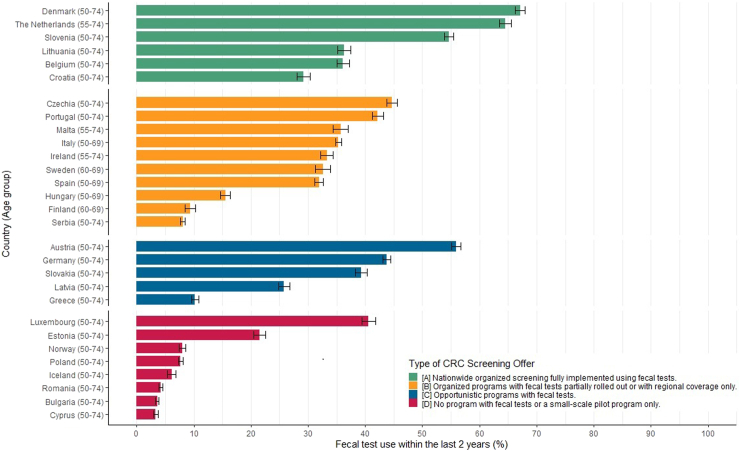
Utilization of fecal occult blood test within the last 2 years by type of CRC screening offer in EHIS wave 3.

Overall, there was wide variation in fecal test utilization among the participating countries and age groups, ranging from 3.3% in Cyprus to as high as 67.1% in Denmark (Fig. 2). Among countries that had fully rolled out their screening programs with fecal tests, more than half of the eligible population had done fecal testing within the preceding two years in Denmark (67.1%), the Netherlands (64.5%), and Slovenia (54.6%). In Lithuania, Belgium, and Croatia, fecal test utilization was much lower (36.3%, 36.1%, and 29.2%, respectively). Countries whose screening programs were still being rolled out nationally had utilization rates ranging from 8.1% in Serbia to 44.7% in Czechia. Among countries offering fecal tests in a predominantly opportunistic manner, utilization rates ranged considerably, from only 10.2% in Greece to 55.9% in Austria. Considerable variation was also observed among countries running small-scale pilot or no programs at all, but generally utilization was lowest in this category (below 10% in all countries except Luxembourg and Estonia).

**Fig. 3:**
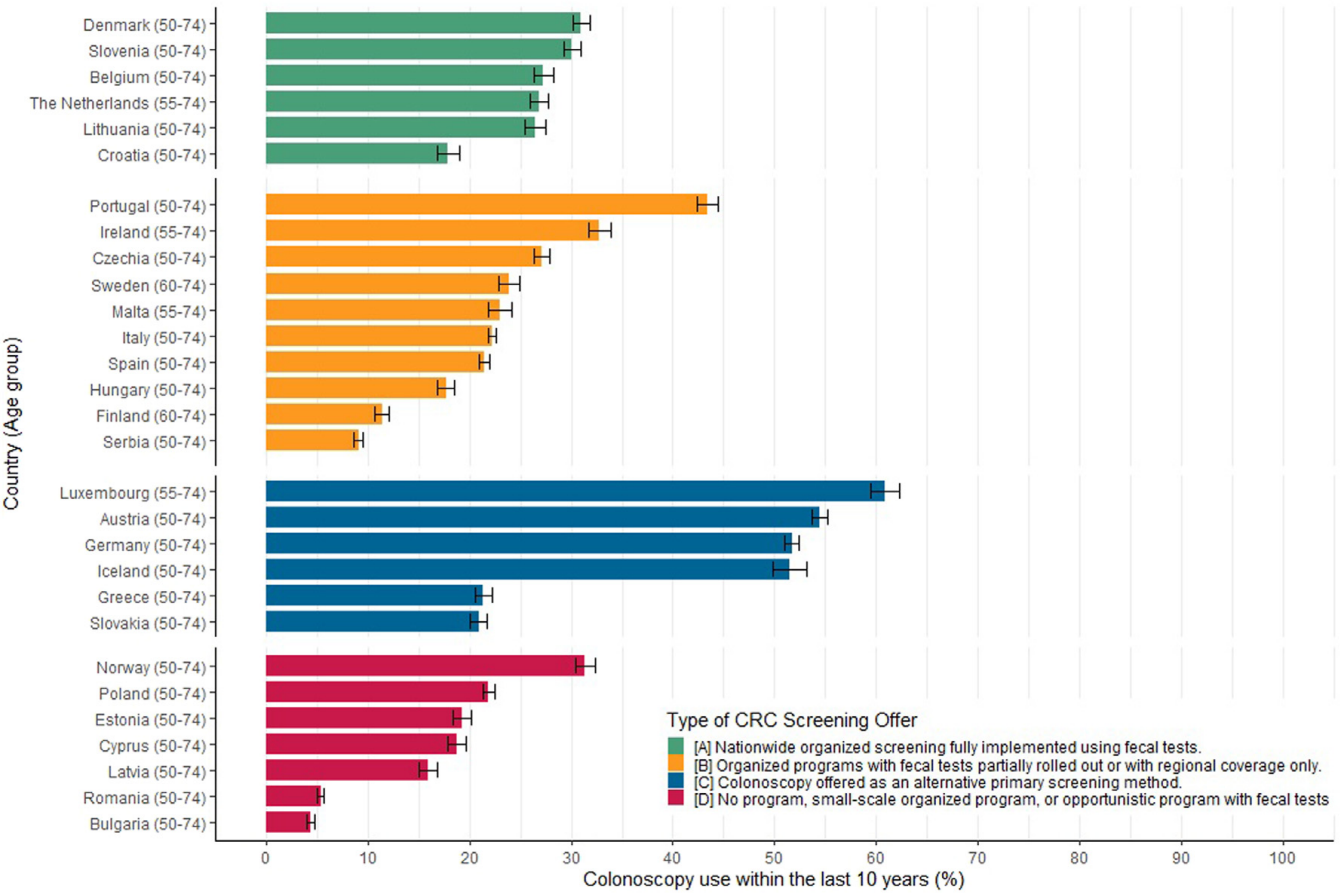
Utilization of colonoscopy within the last 10 years by type of CRC screening offer in EHIS wave 3.

As for the countries offering colonoscopy as an alternative primary screening test, utilization in the preceding 10 years ranged between one-half and almost two-thirds among recommended age groups in all countries (Luxembourg; 60.8%; Austria; 54.4%; Germany; 51.7%; and Iceland; 51.5%), except Greece (21.5%) and Slovakia (20.9%) (Fig. 3). In the remaining countries, use of colonoscopy was overall much lower (below 35% in all countries except Portugal), but again with major variation between those countries.

**Fig. 4:**
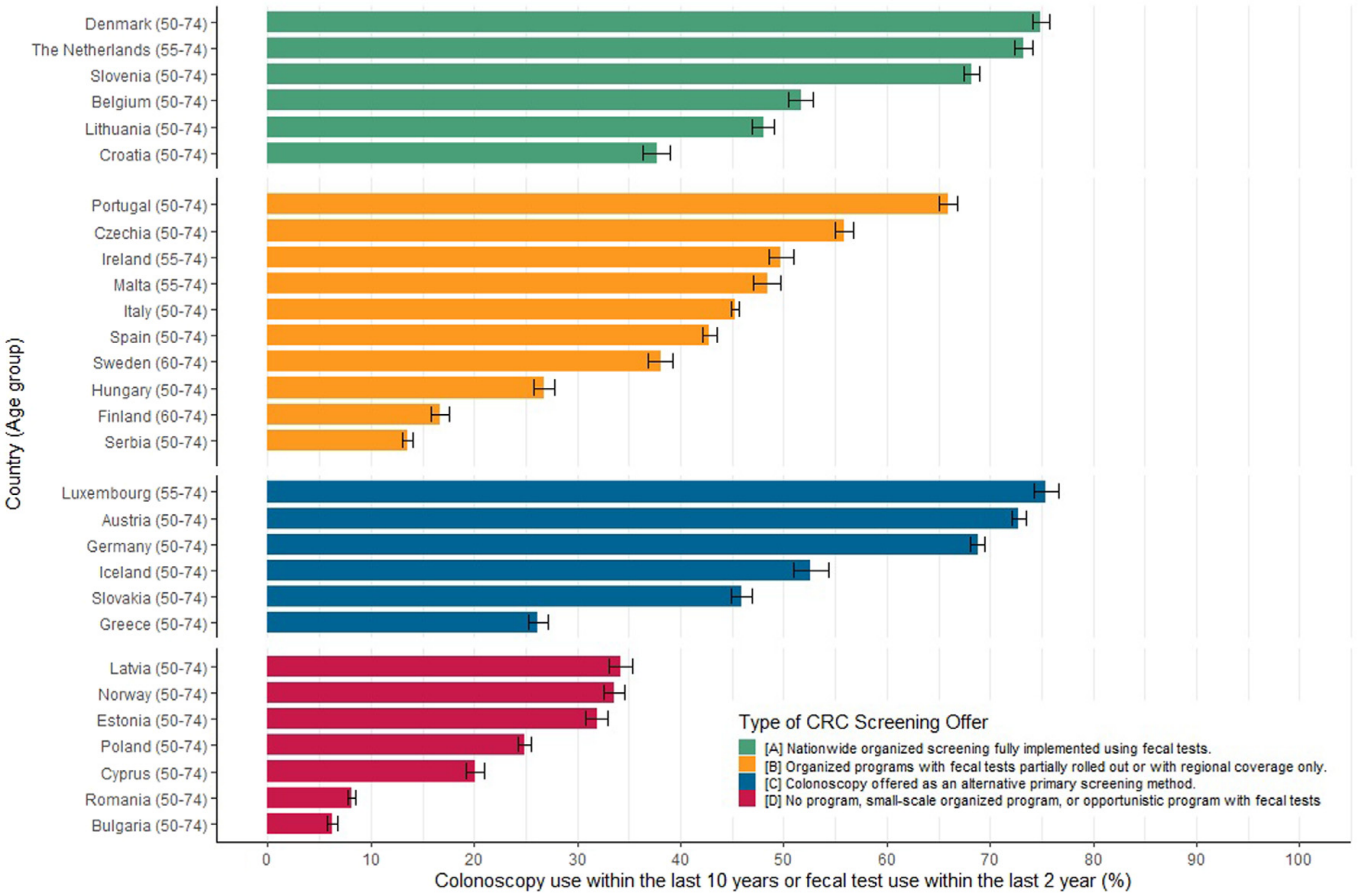
Utilization of fecal tests within the last 2 years or colonoscopy within the last 10 years in EHIS 3.

Overall utilization of fecal tests and/or colonoscopy was higher in countries with fully rolled out organized programs and those offering colonoscopy as a primary screening alternative (above 50% in most countries) and was generally lower (less than 35%) in countries where no program existed (Fig. 4).

### Factors associated with use of CRC screening

Associations of various factors, including sociodemographic and lifestyle factors and healthcare use, with CRC screening test use are shown, according to type of screening offers, in Supplementary Tables S3–S5. Compared to people aged 60–64, younger people aged 50–59 were less likely to use CRC screening tests across all categories. People with less than secondary level education showed up to 21–40% lower odds of using either test compared to those with tertiary level education [ORs ranging from 0.60 (95% CI: 0.47, 0.73) to 0.79 (95% CI: 0.59, 1.00)]. Relative to married and registered partners, those without partners showed nearly 50% lower test usage. Residents of households with three or more people were less likely to have had either of the tests [ORs ranging from 0.81 (95% CI: 0.69, 0.93) among countries with fully rolled-out programs to 0.88 (95% CI: 0.83, 0.92) among those with partially rolled-out programs]. Furthermore, irrespective of the type of screening offered, having a less healthy lifestyle (lower HLS) was associated with lower use of screening. Regarding healthcare use, individuals who have not consulted a physician in over a year were substantially less likely to undergo any CRC screening test, with ORs ranging from 0.45 (95% CI: 0.37, 0.53) to 0.64 (95% CI: 0.54, 0.73) across all types of screening offers.

Living in a rural area was associated with lower use of colonoscopy [ORs 0.76 (95% CI: 0.56, 0.96) to 0.87 (95% CI: 0.81, 0.93)], whereas those who perceived their health to be “less than good” were more likely to have had a colonoscopy, with ORs ranging from 1.16 (95% CI: 1.04, 1.29) to 1.29 (95% CI: 1.18, 1.41) (Supplementary Table S4).

## Discussion

In this study, we assessed the utilization rates of fecal tests and colonoscopy among average-risk adults aged 50–74, along with factors associated with their use, as reported in the European Health Interview Survey 2018–2020 in 29 European countries with different types of screening programs offered. With substantial variations at the national level, only a few countries exceeded at least a 65% utilization threshold with either test (FOBT/FIT in the last 2 years or colonoscopy in the last 10 years) among the eligible population. The overall utilization rates were below 50% in most countries, and the lowest utilization rates were mostly seen in countries with no screening programs. Use of screening offers was furthermore strongly associated with sociodemographic characteristics, with particularly low use among people not living with a partner, living in rural areas, and not having had contact with a physician in the last 12 months.

Among countries with fully implemented organized national programs, Denmark and the Netherlands showed the highest utilization rates, consistent with increasing participation in CRC screening invitations since the launch of the nationwide programs.^15^, ^16^, ^17^ The utilization of fecal tests in Slovenia appears consistent with an earlier estimate, whereas utilization significantly increased in Lithuania compared to findings in EHIS-2.^6^ The full coverage of the Flanders region, which represents up to 57% of the Belgian population, most likely explains the increased uptake of fecal tests in Belgium compared to data from EHIS-2.^6^^,^^18^

Among countries where organized programs were partially rolled out, utilization rates were relatively lower, reflecting variations in demographic coverage, including coverage of much more limited age categories, as previously documented.^6^ In Sweden, two of its three major regions (Norrland and Svealand) had yet to institute any CRC screening system, whereas Finland only covered a few municipalities in an ongoing organized program running at the time of the EHIS survey.^19^^,^^20^ CRC screening in Serbia was relatively new, being introduced barely 4–5 years before the EHIS-3 survey. However, the relatively high participation rates among screening invitees reported for Serbia^21^ and some of the other countries in this category are consistent with an expectation of high utilization when the programs cover all the eligible age groups nationally.

In the few countries where both fecal tests and colonoscopy were offered as alternative primary CRC screening exams in an opportunistic framework, an appreciable rate of utilization of both tests was observed, notably for colonoscopy. Most countries in this group (Germany, Austria, and Luxembourg) have long-running programs^8^ with substantial baseline community awareness about CRC screening. The offering of colonoscopy as an alternative screening method went along with higher overall utilization,^22^ perhaps due to increased propensity for referral by healthcare practitioners. However, utilization rates continued to be low in Greece, which is consistent with findings from previous estimates.^6^ This observation may reflect a constellation of adverse factors, including the failure to implement any of the European Commission’s CRC screening guidelines, the lack of a national cancer strategy, screening plan, cancer registry, and inadequate primary healthcare services.^23^

A wide variation in fecal test utilization was seen among countries where there was no program or only small-scale pilots with fecal tests. Of particular note is the persistently poor rate of utilization in Cyprus, Bulgaria, and Romania, which are countries with no screening programs.^24^, ^25^, ^26^ Although Poland offered colonoscopy-based alternative primary screening, the rate of utilization of colonoscopy was rather low, probably due to its very limited geographic coverage and availability to only individuals aged 55–64 years.^27^

The combined estimates of use of either colonoscopy or fecal testing provided an estimate of the proportion of the eligible population that was up-to-date with screening and showed that much progress and efforts are needed to increase CRC screening use.

Generally, disparities in CRC screening test utilization are observable between countries which implemented population-based programs and those without programs or with small-scale pilots, consistent with findings from prior research.^6^ The failure or delay in establishing population-based screening programs can be attributed to various factors. These may include, but are not limited to, the lack of political will, insufficient allocation of public health resources, challenges in maintaining and sustaining existing screening initiatives, and deficiencies in secondary-level resources, such as endoscopy facilities and trained endoscopists which can impede the overall effectiveness and benefits of screening programs.^23^, ^24^, ^25^, ^26^ The differences in utilization rates among countries with population-based screening programs may also stem from many factors. In particular, screening coverage and organizational characteristics of screening implementation may play significant roles. Additionally, factors such as adoption rates of other cancer screening tests, particularly for breast cancer,^7^^,^^28^ varying levels of population awareness, attitudes toward preventive services, and cultural factors may also play relevant roles.^7^ Apparently, countries with a few high-performing regional screening programs, such as Spain, Sweden, and Italy, exhibited relatively lower average performance compared to those with more consistent coverage across regions. For instance, in 2019, the Italian region of Veneto showed a CRC screening utilization rate exceeding 75%, contrasting starkly with the utilization rate observed in the Puglia region during the same period, which was over six times lower.^29^ This wide discrepancy underscores the urgent imperative to address regional inequalities and expand screening coverage to encompass all eligible individuals across these countries.

In comparison to estimates from other countries or continents where CRC screening tests are also widely available, for example in the United States,^30^ the overall utilization rate aligns with the highest rates we observed in Denmark, the Netherlands, Luxembourg, and Austria. These countries, however, outperformed the highest estimates observed in South Korea in 2020, a country with relatively high utilization rate in Asia.^31^ In Denmark and the Netherlands, utilization is predominantly driven by fecal tests, whereas in Luxembourg and Austria, which largely operate opportunistic programs with colonoscopy as the primary screening method, the rising utilization is predominantly colonoscopy-driven, mirroring the approach and trends observed in the United States.^30^

The identified factors associated with the use of CRC screening tests in this study are in line with findings from previous studies.^6^^,^^17^^,^^22^^,^^32^^,^^33^ As demonstrated in a large Danish study, the odds of non-participation in CRC screening were highest among the youngest eligible age group, immigrants, less educated, or without a partner.^17^ These sociodemographic and socioeconomic characteristics are likely to be related to health-seeking habits, accessibility, affordability, and overall awareness of beneficial health promotion services.

Consistent with our finding, the positive association of poor self-perceived health with CRC screening use had been previously described.^6^^,^^34^ Rural residence has been shown to limit access to several enabling resources for preventive services like screening colonoscopy, including poor awareness of colonoscopy examinations and inadequate access to endoscopy facilities and specialists.^35^

Finally, when specifically compared with findings from EHIS wave 2 analyses,^6^ this study reveals notable changes in screening programs and progress in test utilization across many European countries. Four countries (Denmark, the Netherlands, Lithuania, and Belgium) have achieved national coverage from their previous partial rollout or regional coverage, while Portugal and Hungary have respectively expanded coverage from opportunistic programs and no program or small-scale pilot to partial rollout or regional programs. Similarly, while only Germany, Austria, the United Kingdom, and Slovenia reached or exceeded the 65% up-to-date utilization threshold during EHIS-2,^6^ seven countries (Luxembourg, Denmark, Austria, The Netherlands, Germany, Slovenia, and Portugal) exceeded this threshold in the current analysis. Notwithstanding, achieving the minimum 65% utilization rate as envisioned by the EU Commission^36^ continues to elude the majority of the countries. Our findings also underscore additional factors that have likely gained significance in light of the transitions observed between EHIS-2 and EHIS-3. With the expansion of screening programs to broader populations, other indicators of socioeconomic disparities, including educational attainment, residential location, household size, and marital status, have emerged as significant determinants. This highlights the importance of addressing these variables as countries enhance their screening programs and broaden population coverage.

### Study strengths and limitations

This study used a large population-based data source that included representative samples from all EU countries and beyond. The large number of countries and the heterogeneity of screening offers enabled assessment of screening use across a large variety of health care systems and stages of screening implementation. Furthermore, the overall large sample size allowed to assess factors associated with screening use with adequate power. The identified patterns may provide countries at various stages of CRC screening implementation important information on their achievements in the framework of an international comparison and clues for potential further steps and needs of improvements on the way to achieving cancer screening plans.

A number of limitations are also worth considering, especially in the interpretation and application of the study’s findings. Risk of recall and reporting biases might have led to over- or under-reporting of test use because the data was based on self-reported responses. However, self-reporting of health information, especially cancer-related data, has been shown to demonstrate strong reliability.^37^ The risk of reporting bias was further minimized in this study by eliminating all data provided by a third party. Furthermore, the questions on CRC screening in the EHIS survey did not specifically differentiate between screening and diagnostic testing, especially for colonoscopy, which may have been offered as a follow-up to a positive fecal test. Thus, the estimates in this study could be a mix of screening and diagnostic test use. A further limitation in this regard is the likely imprecision regarding the 10-year time window for analysis of colonoscopy, especially in countries that have newly implemented national screening programs, as it is possible that only few individuals could have had the screening prior to the commencement of such programs in their country. Exposure bias may also explain the lower use rates of colonoscopy within 10 years in the age group 50–59, as screening colonoscopy is not commonly offered before age 50, which implies that participants in this age group could have been eligible for screening colonoscopy for shorter time intervals only.

Even though classification of countries by type of screening programs may have helped to disclose relationships between the kind of programs and screening use, remaining substantial heterogeneity in program features within categories, as reflected in the presented individual-country results, need to be kept in mind. While the EHIS’ focus on mental health symptoms within the preceding two weeks provides valuable insights, it may not capture the comprehensive impact of mental illnesses on CRC screening utilization. Moreover, it is essential to acknowledge that potential biases may still persist in the estimation of individual-level factors associated with screening utilization, considering the diverse nature of the self-reported data used in this study. In particular, retrospective ascertainment of the covariates after the considered periods of screening use precludes establishing temporal relationships for covariates that may have changed during those periods.

### Conclusions

Despite undisputed progress in CRC screening, our study demonstrates that in only a few countries in Europe a majority of the eligible population was up-to-date with fecal testing or colonoscopy. Overall, organized screening programs with fecal tests, especially when all eligible groups are covered, achieved the highest utilization of the screening tests. However, even within such programs, there is a large heterogeneity in screening use, pointing to the critical role of specific features of implementation, such as outreach, screening communication, easy access (e.g. by direct mailing of fecal tests), screening reminders etc. Particular emphasis should be paid to efforts to overcome the screening paradoxon, i.e. the strongest underuse of screening by those with unhealthy lifestyles and highest CRC risk. Enhanced efforts to increase use of CRC screening will be needed to attenuate the strong increase in numbers of CRC cases and deaths that is otherwise to be expected due to the demographic development in the years and decades to come.^38^

## Contributors

Conceptualization of topic: I.O., R.C., and H.B.; Research methodology: I.O., R.C., M.H., and H.B. Data curation: I.O.; data analysis: I.O.; investigation: I.O.; resources: H.B. Writing—Original draft preparation: I.O.; Writing—Co-authors review and editing: I.O., R.C., M.H., and H.B.; project administration: H.B.; Supervision: H.B. All authors have read and approved the submitted version.

## Data sharing statement

Access to study data was granted by Eurostat. Information on how to access the data can be found at https://ec.europa.eu/eurostat/web/microdata/european-health-interview-survey.

## Declaration of interests

The authors declare no commercial or financial relationships that could be construed as a potential conflict of interest in the conduct of this study.

## Appendix ASupplementary data

Supplementary Tables S1–S5 and Item S6
