# Correction to “Utilization of colorectal cancer screening tests across European countries: a cross-sectional analysis of the European health interview survey 2018–2020” [The Lancet Regional Health—Europe Volume 41, June 2024, 100920]

**DOI:** 10.1016/j.lanepe.2025.101287

**Published:** 2025-03-26

**Authors:** Idris Ola, Rafael Cardoso, Michael Hoffmeister, Hermann Brenner

**Affiliations:** a Division of Clinical Epidemiology and Aging Research, German Cancer Research Center (DKFZ), Heidelberg 69120, Germany; b Medical Faculty Heidelberg, University of Heidelberg, Heidelberg 69120, Germany; c Division of Preventive Oncology, German Cancer Research Center (DKFZ) and National Center for Tumor Diseases (NCT), Heidelberg 69120, Germany; d German Cancer Consortium (DKTK), German Cancer Research Center (DKFZ), Heidelberg 69120, Germany

The second author of the paper (Rafael Cardoso) declares being in an editorial capacity at Elsevier in *The Lancet Regional Health*—*Europe* at the time the paper was accepted for publication. Rafael Cardoso had no role in the editorial evaluation, peer-review process, or the decision to accept the paper.

