# Supplementary material for: Utilization of colorectal cancer screening tests across European countries: a cross-sectional analysis of the European health interview survey 2018–2020
Source: Lancet Reg Health Eur. 2024 Apr 29;41:100920. doi: 10.1016/j.lanepe.2024.100920 (PMC11067466; doi:10.1016/j.lanepe.2024.100920)
Supplement: Supplementary Tables S1–S5 and Item S6 [file mmc1.docx]

## **SUPPLEMENTARY MATERIALS**

**Table of contents**

**Page**

Table of contents 1

Table S1. Derivation of the healthy lifestyle score 2

Table S2. Classification of countries by type of CRC screening offer. 3

Table S3. Odds ratios and 95% CIs from subgroup meta-analyses (random effects) of the association between sociodemographic, health-related, and healthcare use factors, and fecal test use within 2 years, by type of CRC screening offer. 4-5

Table S4. Odds ratios and 95% CIs from subgroup meta-analyses (random effects) of the association between sociodemographic, health-related, and healthcare use factors, and colonoscopy use within 10 years, by type of CRC screening offer. 6-7

Table S5. Odds ratios and 95% CIs from subgroup meta-analyses (random effects) of the association between sociodemographic, health-related, and healthcare use factors, and use of fecal tests within 2 years or colonoscopy within 10 years, by type of CRC screening offer. 8-9

Item S6. Reference list for Table 2. 10-13

# Table S1. Derivation of the healthy lifestyle score.

| Lifestyle score | Point allocation | Description |
| --- | --- | --- |
| Body mass index (BMI)^a^ | 0 | Overweight or obese (BMI ≥25 kg/m^2^) |
|  | 1 | Normal weight (>18·5 kg/m2 and <25 kg/m^2^) |
| Physical activity^b^ | 0 | <150 minutes per week |
|  | 1 | ≥150 minutes per week |
| Smoking^c^ | 0 | Daily smoking |
|  | 1 | Nonsmoking or occasional smoking |
| Alcohol^d^ | 0 | ≥2 drinks per day |
|  | 1 | <2 drinks per day |
| Diet | n.a. | n.a. |

# ^a^ BMI = weight (kg)/height (m^2^). In EHIS 3, BMI was computed from weight without clothes and shoes and height without shoes. Underweight respondents (BMI <18.5 kg/m^2^) were not included in the lifestyle score. ^b^ Estimated in hours and minutes as the time spent on doing sports, fitness, or recreational (leisure) physical activities per week. ^c^ Frequency of tobacco smoking. ^d^ Number of alcoholic (standard) drinks on average on any of the days of the week. n.a. = not available.

# Table S2. Classification of countries by type of CRC screening offer

| Analysis of fecal test use | | | | Analyses of colonoscopy use and use of either test. | | | |
| --- | --- | --- | --- | --- | --- | --- | --- |
| Category | Type of CRC screening offer | Countries | Age group (years) | Category | Type of CRC screening offer | Countries | Age group (years) |
| A | Nationwide organized screening fully implemented using fecal tests | Belgium | 50-74 | A | Nationwide organized screening fully implemented using fecal tests | Belgium | 50-74 |
|  |  | Croatia | 55-74 |  |  | Croatia | 50-74 |
|  |  | Denmark | 50-74 |  |  | Denmark | 50-74 |
|  |  | Lithuania | 50-74 |  |  | Lithuania | 55-74 |
|  |  | Netherlands | 50-74 |  |  | Netherlands | 50-74 |
|  |  | Slovenia | 50-74 |  |  | Slovenia | 50-74 |
|  | | | | | | | |
| B | Organized programs with fecal tests partially rolled out or with regional coverage only. | Czechia | 50-74 | B | Organized programs with fecal tests partially rolled out or with regional coverage only. | Czechia | 50-74 |
|  |  | Finland | 60-69 |  |  | Finland | 60-74 |
|  |  | Hungary | 50-69 |  |  | Hungary | 50-74 |
|  |  | Ireland | 55-74 |  |  | Ireland | 55-74 |
|  |  | Italy | 50-69 |  |  | Italy | 50-74 |
|  |  | Malta | 55-74 |  |  | Malta | 55-74 |
|  |  | Portugal | 50-74 |  |  | Portugal | 50-74 |
|  |  | Serbia | 50-74 |  |  | Serbia | 50-74 |
|  |  | Spain | 50-69 |  |  | Spain | 50-74 |
|  |  | Sweden | 60-69 |  |  | Sweden | 60-74 |
|  | | | | | | | |
| C | Opportunistic programs with fecal test | Austria | 50-74 | C | Colonoscopy offered as an alternative primary screening method. | Austria | 50-74 |
|  |  | Germany | 50-74 |  |  | Germany | 50-74 |
|  |  | Greece | 50-74 |  |  | Greece | 50-74 |
|  |  | Latvia | 50-74 |  |  | Iceland | 50-74 |
|  |  | Slovakia | 50-74 |  |  | Luxembourg | 55-74 |
|  |  |  |  |  |  | Slovakia | 50-74 |
|  | | | | | | | |
| D | No program with fecal tests or a small-scale pilot program only. | Bulgaria | 50-74 | D | No program, small-scale organized program, or opportunistic program with fecal tests. | Bulgaria | 50-74 |
|  |  | Cyprus | 50-74 |  |  | Cyprus | 50-74 |
|  |  | Estonia | 50-74 |  |  | Estonia | 50-74 |
|  |  | Iceland | 50-74 |  |  | Latvia | 50-74 |
|  |  | Luxembourg | 50-74 |  |  | Norway | 50-74 |
|  |  | Norway | 50-74 |  |  | Poland | 50-74 |
|  |  | Poland | 50-74 |  |  | Romania | 50-74 |
|  |  | Romania | 50-74 |  |  |  |  |

**Table S3. Odds ratios and 95% CIs from subgroup meta-analyses (random effects) of the association between sociodemographic, health-related, and healthcare use factors, and fecal test use within 2 years, by type of CRC screening offer.**

| **Characteristics** | **Nationwide organized screening fully implemented using fecal tests.** | **Organized programs with fecal tests partially rolled out or with regional coverage only.** | **Opportunistic programs with fecal tests.** | **No program with fecal tests or a small-scale pilot program only.** | **P _subgroup differences_** |
| --- | --- | --- | --- | --- | --- |
|  | OR (95% CI) | OR (95% CI) | OR (95% CI) | OR (95% CI) |  |
| **Sociodemographic factors** | | | | | |
| **Sex** | | | | | |
| Male | 1·00 | 1·00 | 1·00 | 1·00 | 0·36 |
| Female | 1·13 (0·90, 1·37) | 1·00 (0·9, 1·10) | 1·20 (0·97, 1·43) | 1·04 (0·92, 1·15) |  |
| **Age group (years)** | | | | | |
| 50-54 | **0·67 (0·49, 0·85)** | **0·67 (0·57, 0·77)** | **0·84 (0·73, 0·95)** | **0·58 (0·32, 0·83)** | 0·06 |
| 55-59 | 0·86 (0·66, 1·05) | 0·88 (0·72, 1·05) | 0·97 (0·85, 1·08) | **0·72 (0·47, 0·97)** | 0·30 |
| 60-64 | 1·00 | 1·00 | 1·00 | 1·00 |  |
| 65-69 | 0·99 (0·86, 1·11) | 0·96 (0·86, 1·05) | 1·00 (0·89, 1·12) | 1·12 (0·74, 1·50) | 0·82 |
| 70-74 | **0·81 (0·67, 0·94)** | **0·66 (0·44, 0·88)** | 0·98 (0·84, 1·12) | 0·93 (0·55, 1·32) | 0·09 |
| **Marital Status** | | | | | |
| Married/registered partners | 1·00 | 1·00 | 1·00 | 1·00 |  |
| Never Married | **0·58 (0·50, 0·66)** | **0·78 (0·69, 0·87)** | **0·71 (0·62, 0·79)** | **0·74 (0·52, 0·96)** | 0·01 |
| Widowed or divorced | **0·82 (0·74, 0·90)** | **0·86 (0·80, 0·9)** | **0·79 (0·71, 0·86)** | 0·85 (0·63, 1·08) | 0·52 |
| **Educational Status** | | | | | |
| Tertiary | 1·00 | 1·00 | 1·00 | 1·00 |  |
| Upper secondary | 0·97 (0·81, 1·13) | 0·91 (0·80, 1·03) | 0·89 (0·78, 1·00) | 0·89 (0·68, 1·10) | 0·89 |
| Less | 0·86 (0·69, 1·03) | **0·79 (0·62, 0·97)** | **0·72 (0·54, 0·90)** | **0·73 (0·47, 0·99)** | 0·71 |
| **Job** | | | | | |
| Employed | 1·00 | 1·00 | 1·00 | 1·00 |  |
| Retired | 0·91 (0·75, 1·06) | 1·09 (0·98, 1·19) | 1·06 (0·88, 1·25) | 0·90 (0·74, 1·07) | 0·13 |
| Unemployed and others. | **0·84 (0·74, 0·95)** | 0·98 (0·79, 1·17) | 0·89 (0·64, 1·15) | 1·21 (0·98, 1·44) | 0·04 |
| **Location of residence ^a^** | | | | | |
| City | 1·00 | 1·00 | 1·00 | 1·00 |  |
| Town or suburb | 1·04 (0·91, 1·17) | 0·95 (0·73, 1·17) | 1·16(0·61, 1·72) | 0·87(0·66, 1·08) | 0·52 |
| Rural area | 0·99 (0·80, 1·18) | 0·87 (0·69, 1·05) | 0·95 (0·68, 1·22) | **0·81 (0·63, 0·99)** | 0·55 |
| **Citizenship ^b^** | | | | | |
| Natives | 1·00 | 1·00 | 1·00 | 1·00 |  |
| Non-natives | **0·54 (0·39, 0·70)** | **0·65 (0·45, 0·84)** | 0·78 (0·52, 1·05) | 0·84 (0·66, 1·02) | 0·08 |
| **Level of income ^c^** | | | | | |
| Quintiles 4 and 5 | 0·98 (0·87, 1·08) | 1·08 (0·97, 1·19) | 1·03 (0·92, 1·14) | 0·92 (0·72, 1·12) | 0·45 |
| Quintile 3 | 1·00 | 1·00 | 1·00 | 1·00 |  |
| Quintiles 1 and 2 | **0·87 (0·76, 0·98)** | 0·91 (0·82, 1·01) | 0·97 (0·88, 1·07) | 0·94 (0·70, 1·18) | 0·60 |
| **Household size** | | | | | |
| 2 people or less | 1·00 | 1·00 | 1·00 | 1·00 |  |
| 3 people or more | 0·86 (0·72, 1·00) | **0·91 (0·86, 0·96)** | 0·88 (0·73, 1·03) | 0·89 (0·77, 1·01) | 0·91 |
| **Health-related factors** | | | | | |
| **Self-perceived health** | | | | | |
| Good or very good | 1·00 | 1·00 | 1·00 | 1·00 |  |
| Less than good | 0·90 (0·79, 1·00) | 1·01 (0·92, 1·11) | 1·05 (0·92, 1·17) | 1·08 (0·94, 1·23) | 0·24 |
| **Health-related limitation** | | | | | |
| No limitation | 1·00 | 1·00 | 1·00 | 1·00 |  |
| Any limitation | 0·97 (0·80, 1·13) | 0·97 (0·87, 1·07) | 1·04 (0·95, 1·13) | 1·11 (0·96, 1·26) | 0·41 |
| **Mental health problems (last 2 weeks) ^d^** | | | | | |
| No | 1·00 | 1·00 | 1·00 | 1·00 |  |
| Yes | 1·02 (0·82, 1·22) | 1·00 (0·95, 1·06) | **0·91 (0·84, 0·99)** | 1·14 (1·00, 1·28) | 0·03 |
| **Healthy lifestyle score** | | | | | |
| 0 or 1 | **0·88 (0·81, 0·95)** | 0·90 (0·80, 1·00) | **0·79 (0·73, 0·85)** | **0·88 (0·78, 0·98)** | 0·13 |
| 2 | 1·00 | 1·00 | 1·00 | 1·00 |  |
| 3 or 4 | 1·13 (1·00, 1·27) | **1·29 (1·16, 1·42)** | 1·08 (0·90, 1·25) | 1·14 (0·93, 1·35) | 0·20 |
| **Factors related to healthcare Use** | | | | | |
| **Last time of medical consultation with a GP** | | | | | |
| <12 months | 1·00 | 1·00 | 1·00 | 1·00 |  |
| ≥12 months | **0·60 (0·44, 0·76)** | **0·57 (0·46, 0·68)** | **0·44 (0·28, 0·59)** | **0·45 (0·36, 0·54)** | 0·19 |
| **Last time of medical consultation with a medical/Surgical specialist** | | | | | |
| <12 months | 1·00 | 1·00 | 1·00 | 1·00 |  |
| ≥12 months | **0·71 (0·58, 0·84)** | **0·61 (0·49, 0·73)** | **0·61 (0·51, 0·71)** | **0·52 (0·36, 0·68)** | 0·34 |

^a^ Data on location of residence was missing for all respondents from Serbia. ^b^ Citizenship status was removed in the analysis for Norway, Poland, Romania, and Bulgaria due to missing data for all respondents from Norway and a very small sample size for non-natives in the other countries mentioned. ^c^ Based on self-reported disposable household income. ^d^ There was no data on mental health problems for Spain (50-69), thus it was removed from the analysis. Mental health problems was dichotomized based on responses to presence or absence of any symptoms (within last 2 weeks) including loss of interest or pleasure in doing things, depression or feeling hopeless, poor sleep or over-sleeping, loss of energy, poor appetite or overeating, low self-esteem or negative feeling towards self, poor concentration, and anxiety.

CI, confidence intervals. Statistically significant results are in bolded fonts.

**Table S4. Odds ratios and 95% CIs from subgroup meta-analyses (random effects) of the association between sociodemographic, health-related, and healthcare use factors, and colonoscopy use within 10 years, by type of CRC screening offer.**

| **Characteristics** | **Nationwide organized screening fully implemented using fecal tests.** | **Organized programs with fecal tests partially rolled out or with regional coverage only.** | **Colonoscopy offered as an alternative primary screening method.** | **No program with fecal tests or a small-scale pilot program only.** | **P _subgroup differences_** |
| --- | --- | --- | --- | --- | --- |
|  | OR (95% CI) | OR (95% CI) | OR (95% CI) | OR (95% CI) |  |
| **Sociodemographic factors** | | | | | |
| **Sex** | | | | | |
| Male | 1·00 | 1·00 | 1·00 | 1·00 | <0·0001 |
| Female | **0·91 (0·83, 0·99)** | **0·88 (0·83, 0·92)** | 0·97 (0·88, 1·06) | 1·07 (0·97, 1·17) |  |
| **Age** |  |  |  |  |  |
| 50-54 | **0·66 (0·53, 0·78)** | **0·63 (0·57, 0·70)** | **0·52 (0·45, 0·59)** | **0·60 (0·48, 0·72)** | 0·09 |
| 55-59 | **0·76 (0·66, 0·86)** | **0·87 (0·79, 0·94)** | **0·78 (0·68, 0·88)** | **0·71 (0·58, 0·85)** | 0·14 |
| 60-64 | 1·00 | 1·00 | 1·00 | 1·00 |  |
| 65-69 | 1·03 (0·89, 1·16) | 1·10 (1·00, 1·21) | 1·03 (0·85, 1·20) | 1·06 (0·79, 1·34) | 0·80 |
| 70-74 | 1·12 (0·94, 1·30) | **1·17 (1·02, 1·32)** | **1·29 (1·04, 1·54)** | 1·19 (0·88, 1·50) | 0·76 |
| **Marital status** |  |  |  |  |  |
| Married/registered partners | 1·00 | 1·00 | 1·00 | 1·00 |  |
| Never Married | **0·72 (0·61, 0·83)** | **0·78 (0·66, 0·89)** | **0·66 (0·56, 0·76)** | **0·62 (0·42, 0·83)** | 0·43 |
| Widowed or divorced | 1·04 (0·94, 1·15) | 0·96 (0·86, 1·06) | **0·86 (0·78, 0·94)** | 0·88 (0·74, 1·02) | 0·04 |
| **Educational status ^a^** | | | | | |
| Tertiary | 1·00 | 1·00 | 1·00 | 1·00 |  |
| Upper secondary | 0·97 (0·88, 1·07) | **0·87 (0·80, 0·94)** | 0·86 (0·67, 1·05) | **0·80 (0·69, 0·92)** | 0·15 |
| Less | 0·90 (0·73, 1·07) | **0·71 (0·65, 0·78)** | 0·87 (0·62, 1·12) | **0·64 (0·48, 0·79)** | 0·09 |
| **Employment** | | | | | |
| Employed | 1·00 | 1·00 | 1·00 | 1·00 |  |
| Retired | **1·31 (1·13, 1·49)** | **1·22 (1·11, 1·32)** | 1·23 (0·97, 1·48) | 1·03 (0·78, 1·28) | 0·36 |
| Unemployed and others. | 1·10 (0·94, 1·25) | 1·17 (0·99, 1·34) | 1·04 (0·80, 1·28) | **1·28 (1·09, 1·47)** | 0·37 |
| **Location of residence ^b^** | | | | | |
| City | 1·00 | 1·00 | 1·00 | 1·00 |  |
| Town or suburb | 0·91 (0·73, 1·08) | **0·90 (0·83, 0·98)** | 0·93 (0·84, 1·02) | 0·95 (0·74, 1·17) | 0·95 |
| Rural area | **0·86 (0·77, 0·95)** | **0·87 (0·81, 0·93)** | **0·76 (0·56, 0·96)** | **0·78 (0·65, 0·91)** | 0·53 |
| **Citizenship ^c^** | | | | | |
| Natives | 1·00 | 1·00 | 1·00 | 1·00 |  |
| Non-natives | 0·81 (0·60, 1·02) | **0·79 (0·65, 0·92)** | 0·69 (0·34, 1·04) | 0·94 (0·45, 1·43) | 0·88 |
| **Level of income ^d^** | | | | | |
| Quintiles 4 and 5 | 1·05 (0·92, 1·18) | 1·08 (1·00, 1·15) | **1·14 (1·03, 1·25)** | 0·96 (0·84, 1·07) | 0·15 |
| Quintile 3 | 1·00 | 1·00 | 1·00 | 1·00 |  |
| Quintiles 1 and 2 | 0·99 (0·88, 1·11) | **0·91 (0·84, 0·97)** | 0·95 (0·85, 1·06) | 1·00 (0·88, 1·12) | 0·43 |
| **Household size** | | | | | |
| <2 people | 1·00 | 1·00 | 1·00 | 1·00 |  |
| ≥3 people | **0·89 (0·79, 0·98)** | **0·89 (0·83, 0·96)** | **0·86 (0·76, 0·97)** | **0·84 (0·74, 0·93)** | 0·79 |
| **Health-related factors** | | | | | |
| **Self-perceived health** | | | | | |
| Good or very good | 1·00 | 1·00 | 1·00 | 1·00 |  |
| Less than good | **1·27 (1·15, 1·40)** | **1·29 (1·18, 1·41)** | **1·17 (1·03, 1·32)** | **1·16 (1·04, 1·29)** | 0·36 |
| **Health-related limitation** | | | | | |
| No limitation | 1·00 | 1·00 | 1·00 | 1·00 |  |
| Any limitation | **1·19 (1·05, 1·33)** | **1·13 (1·05, 1·22)** | 1·01 (0·76, 1·27) | 1·13 (1·00, 1·27) | 0·71 |
| **Mental health problems (last 2 weeks) ^e^** | | | | | |
| No | 1·00 | 1·00 | 1·00 | 1·00 |  |
| Yes | 1·13 (0·92, 1·34) | **1·07 (1·01, 1·14)** | 1·02 (0·94, 1·11) | **1·12 (1·01, 1·23)** | 0·51 |
| **Healthy lifestyle score ^f^** | | | | | |
| 0 or 1 | 0·90 (0·81, 1·00) | **0·82 (0·75, 0·89)** | **0·75 (0·69, 0·80)** | **0·87 (0·79, 0·96)** | 0·01 |
| 2 | 1·00 | 1·00 | 1·00 | 1·00 |  |
| 3 or 4 | 1·08 (0·91, 1·24) | **1·21 (1·06, 1·36)** | 1·08 (0·97, 1·19) | **1·27 (1·05, 1·48)** | 0·28 |
| **Factors related to healthcare Use** | | | | | |
| **Last time of medical consultation with a GP** | | | | | |
| <12 months | 1·00 | 1·00 | 1·00 | 1·00 |  |
| ≥12 months | **0·58 (0·46, 0·70)** | **0·63 (0·55, 0·70)** | **0·50 (0·42, 0·59)** | **0·53 (0·46, 0·60)** | 0·15 |
| **Last time of medical consultation with a medical/Surgical specialist** | | | | | |
| <12 months | 1·00 | 1·00 | 1·00 | 1·00 |  |
| ≥12 months | **0·60 (0·52, 0·67)** | **0·50 (0·46, 0·55)** | **0·53 (0·42, 0·64)** | **0·52 (0·46, 0·57)** | 0·21 |

^a^ Information on tertiary education was not included in the analysis for Iceland and Malta due to a lack of data. ^b^ Data on location of residence was missing for all respondents from Serbia. ^c^ Citizenship status was removed from the analysis for Norway, Croatia, Romania, and Slovakia due to missing data for all respondents from Norway and a very small sample size for non-natives in the other countries mentioned. ^d^ Based on self-reported disposable household income. ^e^ Information on mental health problems was removed for Spain (50-74) due to a lack of data. Mental health problems was dichotomized based on responses to presence or absence of any symptoms (within last 2 weeks) including loss of interest or pleasure in doing things, depression or feeling hopeless, poor sleep or over-sleeping, loss of energy, poor appetite or overeating, low self-esteem or negative feeling towards self, poor concentration, and anxiety. ^f^ Information on HLS “3 or 4” was removed for Finland due to lack of data. CI, confidence intervals. Statistically significant results are in bolded fonts.

**Table S5. Odds ratios and 95% CIs from subgroup meta-analyses (random effects) of the association between sociodemographic, health-related, and healthcare use factors, and use of fecal tests within 2 years or colonoscopy within 10 years, by type of CRC screening offer.**

| **Characteristics** | **Nationwide organized screening fully implemented using fecal tests.** | **Organized programs with fecal tests partially rolled out or with regional coverage only.** | **Colonoscopy offered as an alternative primary screening method.** | **No program with fecal tests or a small-scale pilot program only.** | **P _subgroup differences_** |
| --- | --- | --- | --- | --- | --- |
|  | OR (95% CI) | OR (95% CI) | OR (95% CI) | OR (95% CI) |  |
| **Sociodemographic factors** | | | | | |
| **Sex** | | | | | |
| Male | 1·00 | 1·00 | 1·00 | 1·00 | 0·43 |
| Female | 1·09 (0·89, 1·29) | 1·00 (0·92, 1·08) | 1·04 (0·83, 1·24) | 1·12 (1·00, 1·25) |  |
| **Age** | | | | | |
| 50-54 | **0·60 (0·43, 0·77)** | **0·60 (0·55, 0·66)** | **0·68 (0·58, 0·77)** | **0·59 (0·42, 0·77)** | 0·61 |
| 55-59 | **0·80 (0·66, 0·95)** | **0·84 (0·78, 0·90)** | **0·84 (0·73, 0·94)** | **0·69 (0·49, 0·88)** | 0·51 |
| 60-64 | 1·00 | 1·00 | 1·00 | 1·00 |  |
| 65-69 | 1·04 (0·90, 1·18) | 1·00 (0·93, 1·07) | 1·03 (0·83, 1·23) | 1·05 (0·75, 1·35) | 0·95 |
| 70-74 | 0·94 (0·79, 1·08) | 0·88 (0·72, 1·05) | 1·24 (0·96, 1·51) | 1·14 (0·75, 1·53) | 0·14 |
| **Marital status** | | | | | |
| Married/registered partners | 1·00 | 1·00 | 1·00 | 1·00 |  |
| Never Married | **0·56 (0·44, 0·69)** | **0·76 (0·67, 0·85)** | **0·66 (0·58, 0·75)** | **0·69 (0·50, 0·88)** | 0·09 |
| Widowed or divorced | **0·81 (0·73, 0·89)** | **0·86 (0·80, 0·93)** | **0·82 (0·70, 0·94)** | 0·88 (0·73, 1·02) | 0·72 |
| **Educational status** | | | | | |
| Tertiary | 1·00 | 1·00 | 1·00 | 1·00 |  |
| Upper secondary | 0·93 (0·81, 1·06) | **0·88 (0·78, 0·97)** | 0·86 (0·68, 1·05) | **0·79 (0·68, 0·90)** | 0·39 |
| Less | **0·79 (0·69, 0·89)** | **0·70 (0·58, 0·82)** | 0·79 (0·59, 1·00) | **0·60 (0·47, 0·73)** | 0·13 |
| **Employment** | | | | | |
| Employed | 1·00 | 1·00 | 1·00 | 1·00 |  |
| Retired | 1·05 (0·91, 1·20) | **1·17 (1·08, 1·25)** | 1·15 (0·85, 1·45) | 0·98 (0·79, 1·17) | 0·26 |
| Unemployed and others. | 0·89 (0·78, 1·01) | 1·00 (0·85, 1·15) | 1·04 (0·82, 1·26) | **1·21 (1·05, 1·38)** | 0·02 |
| **Location of residence ^a^** | | | | | |
| City | 1·00 | 1·00 | 1·00 | 1·00 |  |
| Town or suburb | 0·97 (0·84, 1·11) | 0·88 (0·74, 1·03) | 1·05 (0·95, 1·16) | 0·86 (0·70, 1·02) | 0·14 |
| Rural area | 0·89 (0·71, 1·08) | **0·83 (0·68, 0·97)** | **0·65 (0·34, 0·97)** | **0·77 (0·63, 0·90)** | 0·55 |
| **Citizenship ^b^** | | | | | |
| Natives | 1·00 | 1·00 | 1·00 | 1·00 |  |
| Non-natives | **0·57 (0·32, 0·82)** | **0·70 (0·60, 0·80)** | **0·66 (0·39, 0·94)** | 0·78 (0·53, 1·03) | 0·69 |
| **Level of income ^c^** | | | | | |
| Quintiles 4 and 5 | 16·04 (-13·34, 45·42) | 1·09 (1·00, 1·18) | 1·13 (1·00, 1·27) | 0·95 (0·85, 1·05) | 0·06 |
| Quintile 3 | 1·00 | 1·00 | 1·00 | 1·00 |  |
| Quintiles 1 and 2 | 0·91 (0·80, 1·02) | **0·89 (0·82, 0·96)** | 0·96 (0·86, 1·06) | 0·98 (0·87, 1·08) | 0·74 |
| **Household size** | | | | | |
| <2 people | 1·00 | 1·00 | 1·00 | 1·00 |  |
| ≥3 people | **0·81 (0·69, 0·93)** | **0·88 (0·83, 0·92)** | **0·83 (0·74, 0·93)** | **0·85 (0·76, 0·94)** | 0·64 |
| **Health-related factors** | | | | | |
| **Self-perceived health** | | | | | |
| Good or very good | 1·00 | 1·00 | 1·00 | 1·00 |  |
| Less than good | 1·01 (0·87, 1·16) | **1·18 (1·07, 1·29)** | 1·06 (0·95, 1·17) | **1·13 (1·03, 1·24)** | 0·24 |
| **Health-related limitation** | | | | | |
| No limitation | 1·00 | 1·00 | 1·00 | 1·00 |  |
| Any limitation | 0·98 (0·89, 1·08) | 1·01 (0·93, 1·09) | 1·02 (0·82, 1·21) | 1·11 (1·00, 1·22) | 0·35 |
| **Mental health problems (last 2 weeks) ^d^** | | | | | |
| No | 1·00 | 1·00 | 1·00 | 1·00 |  |
| Yes | 1·05 (0·92, 1·18) | **1·08 (1·02, 1·13)** | 0·96 (0·88, 1·05) | **1·14 (1·05, 1·24)** | 0·04 |
| **Healthy lifestyle score ^e^** | | | | | |
| 0 or 1 | **0·82 (0·75, 0·89)** | **0·84 (0·76, 0·91)** | **0·73 (0·66, 0·79)** | **0·85 (0·78, 0·93)** | 0·03 |
| 2 | 1·00 | 1·00 | 1·00 | 1·00 |  |
| 3 or 4 | 1·17 (0·95, 1·38) | **1·36 (1·23, 1·50)** | **1·15 (1·01, 1·28)** | **1·28 (1·08, 1·49)** | 0·12 |
| **Factors related to healthcare Use** | | | | | |
| **Last time of medical consultation with a GP** | | | | | |
| <12 months | 1·00 | 1·00 | 1·00 | 1·00 |  |
| ≥12 months | **0·53 (0·40, 0·66)** | **0·56 (0·47, 0·64)** | **0·45 (0·37, 0·53)** | **0·48 (0·35, 0·60)** | 0·31 |
| **Last time of medical consultation with a medical/Surgical specialist** | | | | | |
| <12 months | 1·00 | 1·00 | 1·00 | 1·00 |  |
| ≥12 months | **0·64 (0·54, 0·73)** | **0·56 (0·50, 0·62)** | **0·52 (0·40, 0·64)** | **0·52 (0·45, 0·60)** | 0·27 |

^a^ Data on the location of residence was missing for all respondents from Serbia. ^b^ Citizenship status was removed from the analysis for Norway and Romania due to missing data for all respondents from Norway and a very small sample size for non-natives in Romania. ^c^ Based on self-reported disposable household income. ^d^ Information on mental health problems was removed for Spain (50-74) due to a lack of data. Mental health problems was dichotomized based on responses to presence or absence of any symptoms (within last 2 weeks) including loss of interest or pleasure in doing things, depression or feeling hopeless, poor sleep or over-sleeping, loss of energy, poor appetite or overeating, low self-esteem or negative feeling towards self, poor concentration, and anxiety. ^e^ Information on HLS “3 or 4” was removed for Finland due to a lack of data. CI, confidence intervals. Statistically significant results are in bolded fonts.

**Item S6. Reference list for Table 2.**

1 Tran TN, Peeters M, Hoeck S, Hal GV, Janssens S, De Schutter H*.* Optimizing the colorectal cancer screening programme using faecal immunochemical test (FIT) in Flanders, Belgium from the “interval cancer” perspective. *Br J Cancer* 2022; **126:**1091–99. <https://doi.org/10.1038/s41416-021-01694-2>

2 Belgian Cancer Registry. Contribution of the Belgian cancer registry to screening programs. Available at <https://kankerregister.org/media/docs/academischezitting10.12.15/BCR_PreventionCancerBurden2015_banner.pdf> (Accessed February 18, 2023).

3 United European Gastroenterology. Colorectal cancer screening across Europe. Published March 2019. Available online at ueg.eu/files/779/67d96d458abdef21792e6d8e590244e7.pdf. [Accessed March 20, 2023]

4 Katičić M, Antoljak N, Kujundžić M, et al. Results of National Colorectal Cancer Screening Program in Croatia (2007-2011). *World J Gastroenterol* 2012; **18**:4300–7. doi: 10.3748/wjg.v18.i32.4300.

# 5 Basu P, Ponti A, Anttila A, et al. Status of implementation and organization of cancer screening in The European Union Member States-Summary results from the second European screening report. *Int. J. Cancer* 2018; 142: 44–56.

6 Nielsen JB, Berg-Beckhoff G, Leppin A. To do or not to do - a survey study on factors associated with participating in the Danish screening program for colorectal cancer. *BMC Health Serv Res*. 2021; **21**:43. doi: 10.1186/s12913-020-06023-6.

7 Dulskas A, Poskus T, Kildusiene I, et al. National Colorectal Cancer Screening Program in Lithuania: Description of the 5-Year Performance on Population Level. *Cancers (Basel)* 2021; **13:**1129. doi: 10.3390/cancers13051129.

8 Poskus T, Strupas K, Mikalauskas S, et al. Initial results of the National Colorectal Cancer Screening Program in Lithuania. Eur J Cancer Prev. 2015; **24:**76–80. doi: 10.1097/CEJ.0000000000000096.

9 Breekveldt ECH, Toes-Zoutendijk E, van de Schootbrugge-Vandermeer HJ, et al. Factors associated with interval colorectal cancer after negative FIT: Results of two screening rounds in the Dutch FIT-based CRC screening program. *Int J Cancer* 2023; **152**:1536–46. doi:[10.1002/ijc.34373](https://doi.org/10.1002/ijc.34373)

10 Tepeš B, Bracko M, Novak Mlakar D, et al. Results of the FIT-based National Colorectal Cancer Screening Program in Slovenia. *J Clin Gastroenterol* 2017; **51:** e52–e59. doi: 10.1097/MCG.0000000000000662.

11 The Svit prgramme-10 years of colorectal cancer screening in Slovenia: Compendium on the 10th anniversary of the Svit Programme. National Institute of Public Health, Ljublana, 2019. <https://www.program-svit.si/wp-content/uploads/2019/10/SVIT-10-LET-ANG-Elektronska.pdf> (Accessed February 3, 2023)

12 Cardoso R, Guo F, Heisser T, Hoffmeister M, Brenner H. Utilization of colorectal cancer screening tests in European countries by type of screening offer: Results from the European Health Interview Survey. *Cancers (Basel)* 2020; **12:**1409. doi: 10.3390/cancers12061409.

13 Czech National Cancer Control Programme. Personalised invitations of Czech citizens to cancer screening programmes. Comprehensive Cancer Care Network CanCon. Available at <https://www.onconet.cz/index-en.php?pg=news&aid=987> (Accessed February 17, 2023).

14 Suchanek S, Grega T, Ngo O, et al. How significant is the association between metabolic syndrome and prevalence of colorectal neoplasia? *World J Gastroenterol* 2016; **22**: 8103–11. doi: 10.3748/wjg.v22.i36.8103.

15 Zavoral M, Suchanek S, Majek O, et al. Colorectal cancer screening: 20 years of development and recent progress. *World J Gastroenterol* 2014; **20**: 3825–34. doi: 10.3748/wjg.v20.i14.3825.

16 Sarkeala T, Färkkilä M, Anttila A, et al. Piloting gender-oriented colorectal cancer screening with a faecal immunochemical test: population-based registry study from Finland. *BMJ Open* 2021; **11:** e046667. doi: 10.1136/bmjopen-2020-046667.

17 Färkkilä M, Heinävaara S, Hyöty M, et al. Protocol for colorectal cancer screening. Recommendation of the expert group set up by the National Cancer Screening Steering Group. 2021. <https://syoparekisteri.fi/assets/files/2021/11/Protocol-for-and-tests-used-in-colorectal-cancer-screening.pdf> (Accessed 30 January, 2023)

# 18 Rutka M, Molnár T, Bor R, et al. Populációalapú “pilot” colorectalis rákszűrés eredményessége. Csongrád megye, 2015 [Efficacy of the population-based pilot colorectal screening program. Hungary, Csongrád county, 2015]. *Orv Hetil*. 2017; 158:1658–67. doi: 10.1556/650.2017.30822.

# 19 Csanádi M, Gini A, de Koning H, et al. Modeling costs and benefits of the organized colorectal cancer screening programme and its potential future improvements in Hungary. *J of Med Screening* 2021; 28:268–76. doi:10.1177/0969141320968598

20 BowelScreen Programme Report 2016 – 2017 Round Two. Available online at: <https://www.bowelscreen.ie/_fileupload/Programme%20Reports/BowelScreen-Programme-Report%20-2016-2017-FINAL-WEB-21_01_20.pdf> (Accessed February 14, 2023).

21 McFerran E, Kee F, Coleman HG. Colorectal cancer screening: Surely FIT for us too. *Frontline Gastroenterol.* 2019; **10**:445–446. <http://dx.doi.org/10.1136/flgastro-2018-101125>

22 Battisti F, Falini P, Gorini G, et al. Cancer screening programmes in Italy during the COVID-19 pandemic: an update of a nationwide survey on activity volumes and delayed diagnoses. *Ann Ist Super Sanita*. 2022; **58**:16–24. doi: 10.4415/ANN_22_01_03.

23 Zorzi M, Da Re F, Mantellini P, et al. Screening for colorectal cancer in Italy: 2011-2012 survey. *Epidemiol Prev*. 2015; **39**(3 Suppl 1): 93–107.

24 Schreuders EH, Ruco A, Rabeneck L, et al. Colorectal cancer screening: A global overview of existing programmes. *Gut* 2015; **64**:1637–49. <http://dx.doi.org/10.1136/gutjnl-2014-309086>

25 Government of Malta. Colorectal Screening. National Colorectal Cancer Screening Programme. 2020. <https://deputyprimeminister.gov.mt/en/phc/nbs/Pages/Screening-Programmes/Colorectal-Screening.aspx>**.** (Accessed February 15, 2023)

26 Directorate-General of Health, Portugal. Rastreio Oportunístico do Cancro do Cólon e Reto [Opportunistic Screening for Colorectal Cancer]. Lisbon, Portugal, 2014. Available at <https://www.nghd.pt/uploads/noc_rccr_act.pdf> (Accessed February 16, 2023).

27 Nogueira RA. (Programa Nacional para as Doenças Oncológicas: o despacho n.º 8254/2017, de 21 de setembro, do Secretário de Estado Adjunto e da Saúde, merece mais atenção e representa um primeiro passo para a orientação de soluções)National Program for Oncological Diseases: The Order No. 8254/2017 of September 21, of the Assistant Secretary of State and Health, deserves more attention and represents a first step towards the guidance of solutions. *Portuguese J of Gen and Fam Med*. **34**:104–9. <https://doi.org/10.32385/rpmgf.v34i2.12403>

28 Currais P, Mão de Ferro S, Areia M, Marques I, Mayer A, Dias Pereira A. Should colorectal cancer screening in Portugal start at the age of 45 years? A cost-utility analysis. *GE Port J Gastroenterol.* 2021; **28**:311–18. doi: 10.1159/000513592.

29 Banković-Lazarević D, Krivokapić Z, Barišić G, Jovanović V, Ilić D, Veljković M. Organized colorectal cancer screening in Serbia - the first round within 2013-2014. *Vojnosanit Pregl.* 2016; **73**:360–7. doi: 10.2298/VSP150421113B.

30 Jovanovic V, Bankovic-Lazarevic D. Organized colorectal cancer screening program: experience from Serbia, *COLOMED*, 2017. <https://www.researchgate.net/publication/341152055_Organized_colorectal_cancer_screening_program_experience_from_Serbia_COLOMED_Rome_2017> (accessed February 15, 2023).

31 Trejo DS, Villares IP, Pinol JAE, et al. Implementation of colorectal cancer screening in Spain: Main results 2006-2011. *Eur. J. Cancer Prev*. 2017; **26**:17–26.

32 Network of Cancer Screening Programmes. Evaluación programas de cribado de cancer colorrectal-2017. (2018). Available at <file:///C:/Users/i200a/Downloads/programasdeccr2017.pdf> pg. 5-13. (Accessed February 16, 2023).

33 Senore C, Basu P, Anttila A, et al. Performance of colorectal cancer screening in the European Union Member States: Data from the second European screening report. *Gut* 2019; **68:**1232–44.

34 Lund University Cancer Center. Screening for colorectal cancer starts this spring. Available online at <https://www.lucc.lu.se/article/screening-colorectal-cancer-starts-spring#:~:text=In%20May%2C%20screening%20for%20colorectal%20cancer%20will%20be,lives%20are%20expected%20to%20be%20saved%20per%20year>. (Accessed April 24, 2023)

35 Gsur A, Baierl A, Brezina S. Colorectal Cancer Study of Austria (CORSA): A Population-Based Multicenter Study. *Biology* 2021; **10:**722. https://doi.org/10.3390/ biology10080722

36 Program for the early detection of colorectal cancer. Available at <https://www.g-ba.de/themen/methodenbewertung/ambulant/frueherkennung-krankheiten/erwachsene/krebsfrueherkennung/darmkrebs-screening/> (Accessed February 15, 2023).

37 Guo F, Chen C, Schottker B, Holleczek B, Hoffmeister M, Brenner H. Changes in colorectal cancer screening use after introduction of alternative screening offer in Germany: Prospective cohort study. *Int. J. Cancer* 2020; **146:** 2423–32.

38 Heisser T, Weigl K, Hoffmeister M, Brenner H. Age-specific sequence of colorectal cancer screening options in Germany: A model-based critical evaluation. *PLoS Med.* 2020; **17**: e1003194. doi: 10.1371/journal.pmed.1003194.

39 Mārcis Leja. Organised colorectal cancer screening needed in Latvia. (Presentation at the Cancer Control Joint Action 2014: Available at <https://www.cancercontrol.eu/archived/news/10/26/Organised-colorectal-cancer-screening-needed-in-Latvia/d%2cnews.html> (Accessed February 16, 2023).

# 40 Ricová JT. Súčasný skríning kolorektálneho karcinómu na Slovensku a úloha VLD. (2020). Available online (in Slovak) <https://www.noisk.sk/files/2021/2021-02-10-skrining-kolorektalneho-karcinomu-na-slovensku-a-uloha-vld.pdf> [Accessed February 13, 2023].

41 Tsvetanova DR, Dimitrova DD, Angelova LB, et al. Feasibility of immunochemical faecal occult blood testing for colorectal cancer screening in Bulgaria. *J BUON*. 2015; **20:**413–20.

42 OECD (2023), *EU Country Cancer Profile: Cyprus 2023*, EU Country Cancer Profiles, OECD Publishing, Paris, <https://doi.org/10.1787/86732eb6-en>.

43 Innos K, Reima H, Baburin A, Paapsi K, Aareleid T, Soplepmann J. Subsite- and stage-specific colorectal cancer trends in Estonia prior to implementation of screening. *Cancer Epidemiol.* 2018; **52**:112–19. doi: 10.1016/j.canep.2017.12.016.

44 Reima H, Soplepmann J, Elme A, et al. Changes in the quality of care of colorectal cancer in Estonia: a population-based high-resolution study. *BMJ Open.* 2020; **10**: e035556. doi: 10.1136/bmjopen-2019-035556.

# 45 Guðlaugsdóttir S. Implementing colorectal cancer screening program in Iceland. In: WEO Barcelona October 2015 proceedings of WEO Colorectal Cancer Screening Meeting. Barcelona, Spain. Available from: <https://www.worldendo.org/wp-content/uploads/2016/08/6_sunna_guolaugsdottir_iceland_ueg2015.pdf>

46 Ministry of Health of Luxemboug (Grand-Duché de Luxembourg). Programme de Dépistage Organisé du Cancer ColoRectal Grand-Duché de Luxembourg 2022. Available at <file:///C:/Users/i200a/Downloads/pdoccr-programme-de-depistage.pdf> (Accessed February 17, 2023).

47 Bhargava S, Czapka E, Hofvind S, Kristiansen M, Diaz E, Berstad P. Polish immigrants' access to colorectal cancer screening in Norway - a qualitative study. *BMC Health Serv Res.* 2022; **22**:1332. doi: 10.1186/s12913-022-08719-3.

48 Cancer Registry of Norway. Colorectal Cancer Screening, Bowel Cancer Screening in Norway – A Pilot Study. Available online: <https://www.kreftregisteret.no/en/screening/Screening-for-colorectal-cancer/> (accessed February 10, 2023).

# 49 Giske Ursin. Pilot Study of a National Screening Programme for Bowel Cancer in Norway. ClinialTrials.gov. 2012. <https://beta.clinicaltrials.gov/study/NCT01538550> (accessed on February 10, 2023).

50 Randel KR, Schult AL, Botteri E, et al. Colorectal cancer screening with repeated fecal immunochemical test versus sigmoidoscopy: Baseline results from a randomized trial. *Gastroenterology*. 2021; **160**:1085–96.e5. doi:10.1053/j.gastro.2020.11.037

51 Krzeczewski B, Hassan C, Krzeczewska O, et al. Cost-effectiveness of colonoscopy in an organized screening program. *Pol Arch Intern Med.* 2021; **131**:128–35. doi:10.20452/pamw.15779

52 Wieszczy P, Kaminski MF, Franczyk R, et al. Colorectal Cancer Incidence and Mortality After Removal of Adenomas During Screening Colonoscopies. *Gastroenterology* 2020; **158**:875–83.e5. doi: 10.1053/j.gastro.2019.09.011.

53 Polish Society of Oncology. Cancer control strategy for Poland 2015-2024. (2017). Available online at <https://ligawalkizrakiem.pl/images/content/Strategia-Walki-z-Rakiem-w-Polsce/Strategia_wersja_ang_2017.pdf> (Accessed September 19, 2023).

54 Bărbulescu LN, Mogoantă SȘ, Bărbulescu LF, Kamal C, Popa DL, Popa RT. A pilot colorectal cancer study using fecal occult blood tests and colonoscopy to identify the weaknesses of the romanian public healthcare system before implementing national screening. *Int. J of Envir Res and Public Health* 2023; **20**:2531. <https://doi.org/10.3390/ijerph20032531>
